# A Molecular Modeling Approach to Identify Novel Inhibitors of the Major Facilitator Superfamily of Efflux Pump Transporters

**DOI:** 10.3390/antibiotics8010025

**Published:** 2019-03-15

**Authors:** Sandra G. Zárate, Paula Morales, Katarzyna Świderek, Victor M. Bolanos-Garcia, Agatha Bastida

**Affiliations:** 1 Facultad de Tecnología, Carrera de Ingeniería Química, Universidad Mayor Real y Pontífice de San Francisco Xavier de Chuquisaca, Regimiento Campos 180, Casilla 60-B, Sucre, Bolivia; 2 Instituto de Química Física Rocasolano, c/Serrano 119, E-28006 Madrid, Spain; pmorales@iqfr.csic.es; 3 Department de Química Física i Analítica, Universitat Jaume I, 12071 Castelló, Spain; swiderek@uji.es; 4 Department of Biological and Medical Sciences, Faculty of Health and Life Sciences, Oxford Brookes University, Oxford OX3 OBP, UK; vbolanos-garcia@brookes.ac.uk; 5 Departamento de Química Bio-orgánica, IQOG, c/ Juan de la Cierva 3, E-28006 Madrid, Spain

**Keywords:** NorA efflux pump, major facilitator super family, bioinformatics, molecular docking, *S. aureus*

## Abstract

Multidrug efflux systems play a prominent role in medicine, as they are important contributors to bacterial antibiotic resistance. NorA is an efflux pump transporter from the major facilitator superfamily that expels numerous drug compounds across the inner membrane of *Staphylococcus aureus* (*S. aureus*). The design of novel inhibitors to combat drug efflux could offer new opportunities to avoid the problem of antibiotic resistance. In this study, we performed molecular modeling studies in an effort to discover novel NorA efflux pump inhibitors. A group of over 673 compounds from the PubChem database with a high (>80%) level of similarity to the chemical structure of capsaicin was used to study the binding affinity of small molecule compounds for the NorA efflux pump. Ten potential lead compounds displayed a good druggability profile, with one in particular (CID 44330438) providing new insight into the molecular mechanism of the inhibition of major facilitator superfamily (MFS) efflux pump transporters. It is our hope that the overall strategy described in this study, and the structural information of the potential novel inhibitors thus identified, will stimulate others to pursue the development of better drugs to tackle multidrug resistance in *S. aureus*.

## 1. Introduction

**Scheme 1:**
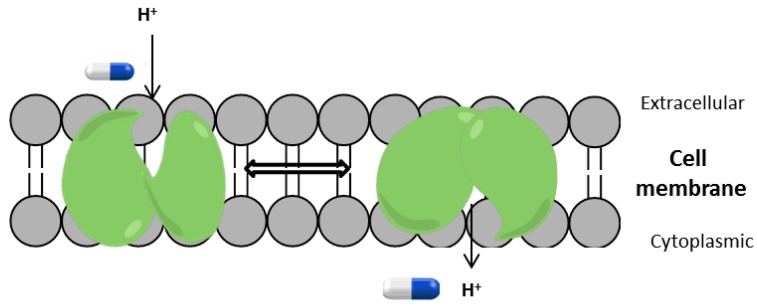
Mechanism for hydrophobic substrate transport by major facilitator superfamily (MFS), “clamp-and-switch model” (Multidrug/H+ antiporter EmrD) [8].

Bacterial resistance to antimicrobial agents is a global problem in medicine [1]. Bacteria can use different mechanisms of resistance including antibiotic receptor alteration, antibiotic modification, and antibiotic efflux by integral membrane transporters [2,3,4]. Multidrug resistance (MDR) transporters, or efflux pumps, play a prominent role in acquired clinical resistance [5,6]. Efflux pump proteins are classified into two families: ATP-binding cassette (ABC) and major facilitator superfamily (MFS). The classification of proteins is based on their energy requirements. ABC protein members directly couple drug efflux to ATP hydrolysis [7], whereas members of the MFS use the proton motive force (PMF) as an energy generator across the cell membrane [8]. MFS proteins are found in all branches of the tree of life and are implicated in a range of diseases, making them important targets for drug discovery. Twenty-three MFS transporter structures have been determined. All share the same structural fold, and they can be divided into three groups: uniporters (transport a single substrate), symporters (transport substrate with protons), and antiporters (transport substrate and co-substrate in opposite directions). MFS transporters move substrates across the inner leaflet of the cell membrane bilayer by a “clamp-and-switch model” (Scheme 1). This mechanism has been proposed for other MDR transporter families that have hydrophobic pockets [8]. 

NorA, from *Staphylococcus aureus*, is a member of the major facilitator superfamily (MFS) and a proton dependent transporter, whose overexpression is associated with drug resistance [9]. Some efflux pump inhibitors (EFIs) can restore the activity of antibiotics by inhibiting the MDR efflux pumps [10,11,12,13,14,15,16,17]. Due to the fact that the NorA efflux pump generates resistance to fluoroquinolones, its inhibition appears attractive for improving potency and enhancing the spectrum of activity of drugs against *S. aureus*.

In silico structure-based screening of inhibitors for MDR efflux pumps can be effective for identifying small molecules that reduce the efflux resistance to quinolones [18,19]. Capsaicin was described recently as a good inhibitor of NorA efflux pumps, because it can reduce the minimum inhibitory concentration (MIC) of ciprofloxacin for *S. aureus* [20].

In an attempt to address the global burden of MDR in *S. aureus*, this paper presents a computer-aided drug identification of potential NorA inhibitors. The NorA 3D structure model was constructed based on the crystal structure of the MFS protein EmrD, and used to perform the molecular docking of 620 compounds deposited in the PubChem library that share 80% or higher similarity to the chemical structure of capsaicin (CID 1548943). In addition to the calculation of interaction energies and in silico pharmacokinetics, promiscuous structural features were considered and filtered out for the selection of potential new lead compounds that target the NorA efflux pump.

## 2. Results and Discussion

### 2.1. Preparation of the NorA Target

NorA MFS from *S. aureus* is a quinolone resistance protein of 388 amino acids, with a molecular mass of 42.32 kDa (Uniprot Q5HHX4) [21]. Multiple sequence alignments suggest that EmrD MFS from *E. coli* (pdb ID: 2GFP) [22] is the closest homolog of NorA, with 16% and 41% amino acid sequence identity and similarity, respectively. The high amino acid sequence similarity between NorA and EmrD from *E. coli* enabled the use of a homology modeling approach to produce a 3D structure model of the former MFS protein. 

**Figure 1:**
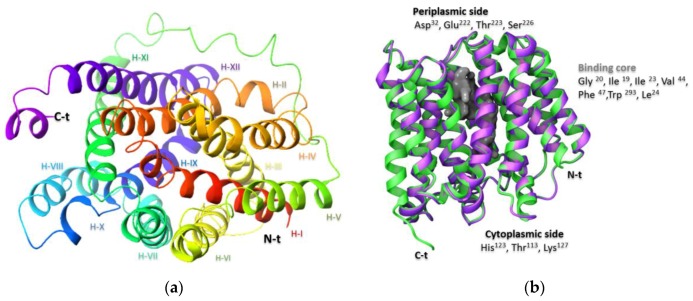
(**a**) 3D model structure of the NorA efflux pump from *S. aureus*, showing that both the N-and C-terminal domains are connected by XII α-helices and cytoplasmic loops. (**b**) Superposition of the 3D structure of the MFS proteins NorA (purple) and EmrD (green). The ligand binding core is highlighted in purple.

The NorA structure model shows a topology consisting of XII transmembrane alpha helices (H-I to H-XII), connected by cytoplasmic loops and two domains; an N-domain (H-I to H-VI) and a C-domain (H-VII to H-XII) (Figure 1a; Supplementary Materials, Table S1 and Figure S1). The quality of the NorA 3D structure model was assessed with the SAVES server [23] (Supplementary Materials, Figure S2).

MFS proteins comprise hydrophobic residues which enable them to export a broad spectrum of lipophilic drugs [24]. Site Map was used to identify the binding core of the NorA model. Three cavities were detected (cytoplasmic side, binding core region, and periplasmic side, Figure 1b and Supplementary Materials Figure S3). The binding core of NorA comprises the hydrophobic residues Ile^19^, Ile^23^, leu^26^, Val^44^, Ser^138^, Trp^293^, and Phe^47^, while the periplasmic and cytoplasmic sides are defined by polar residues such as Asp^32^, Thr^223^, Ser^226^, Glu^222^, Thr^113^, and His^123^, which are highly conserved across the MFS protein family (Supplementary Materials, Table S2) [22]. The substrate binding core, composed mostly of hydrophobic residues, was used to prepare the protein grid. 

### 2.2. Molecular Docking Simulation of NorA Capsaicin and Ciprofloxacin Binding

**Figure 2:**
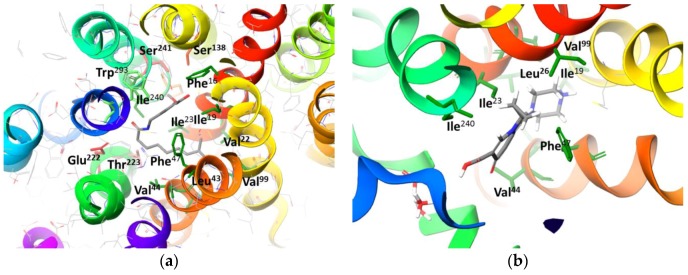
Molecular docking: (**a**) the NorA inhibitor capsaicin and (**b**) the NorA substrate ciprofloxacin. Residues are shown in grey (LeuVal, Phe, Ile, Trp), red (Glu), and orange (Thr, Ser). Figure was generated using the Schrödinger suite.

Firstly, the NorA efflux pump inhibitor capsaicin [20] and the NorA substrate ciprofloxacin (CID 2764) were docked to assess the binding mode of these molecules to the NorA binding pocket. Computational docking analysis showed that both compounds bind to the same hydrophobic cavity. Capsaicin was located closer to the periplasmic side, which is defined by the residues Ser^226^, Glu^222^, and Thr^223^, a finding that provides new molecular insight into the underlying molecular mechanism of efflux pump inhibition [20]. Both the inhibitor and the substrate were able to form nonbonding stabilizing interactions, such as Pi–Pi (T-shape) interactions, with the Trp^293^ and Phe^47^ residues in the case of capsaicin and with the residue Phe^47^ in the case of ciprofloxacin. Additionally, hydrophobic interactions were identified for those compounds involving the NorA residues Val^22^, Ile^23^, Phe^16^, Ile^19^, Val^44^, and Leu^43^ (see Figure 2). Interestingly, the binding energy for capsaicin was estimated to be −7.19 kcal/mol. In contrast, ciprofloxacin showed a lower binding energy of −6.8 kcal/mol. The difference in binding energies can be explained through the analysis of the number of Pi–Pi interactions established between the ligands and the protein hydrophobic pocket, where the formation of two Pi–Pi stacking interactions seemed to contribute to a higher binding affinity compared to a single Pi–Pi stacking interaction. Overall, the predicted binding affinity of ciprofloxacin for NorA was similar to that of other small multidrug resistance (SMS) efflux pumps with quinolones [19]. It does not escape to our attention that the residues Trp^293^, Tyr^131^, and Phe^47^ of NorA could play a key role in the establishment of similar stabilizing interactions with aromatic drugs (Figure S4) [25].

### 2.3. Molecular Docking Simulation of Potential Novel NorA Inhibitors

**Figure 3:**
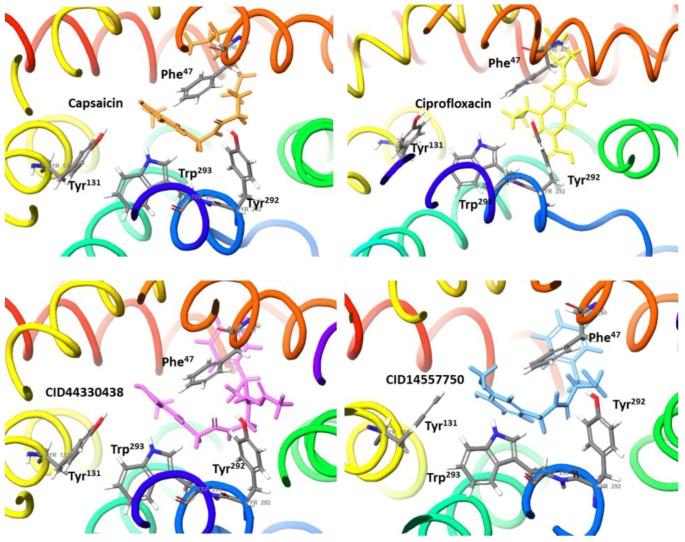
Binding poses of capsaicin (orange), ciprofloxacin (yellow), CID 44330438 (pink), and CID14557750 (blue) within the hydrophobic core (residues in grey) of the NorA efflux pump.

Molecular docking was performed in an attempt to identify novel NorA efflux pump inhibitors of high structure similarity to capsaicin. In this manner, 620 compounds which showed more than 80% structural similarity to capsaicin, and which satisfied Lipinski and Pan Assay Interference compounds (PAINS) rules, were selected and docked into the NorA hydrophobic core. Ten lead compounds with predicted higher binding affinities than capsaicin were selected, and their pharmaceutical properties were evaluated in silico (Supplementary Materials, Table S3). The compounds with the best hits are shown in Table 1. The binding mode of capsaicin, ciprofloxacin, and the two best docked compounds (CID 44330438 and CID 14557750) with the aromatic residues Phe^47^, Tyr^131^, Tyr^292^, and Trp^293^ of NorA are shown in Figure 3.

Table 2 shows the 10 best hits compounds according to qikProp. CID 44330438 and CID 14557750 showed the highest binding energy (−8.14 kcal/mol, −8.02 kcal/mol, respectively) and were predicted to bind the hydrophobic cleft, establishing two Pi–Pi stacking interactions with Phe^47^. All other lead compounds showed a higher affinity than capsaicin and were bound in the same hydrophobic internal pocket. All lead compounds satisfied Lipinski’s rule of five, PAINS, and absorption, distribution, metabolism, excretion, and toxicity filters (ADME/Tox; absorption, distribution, metabolism, excretion, and toxicity). None of the compounds are predicted to exhibit adverse effects (additional pharmacological properties are described in Supplementary Materials, Table S3).

**Figure 4:**
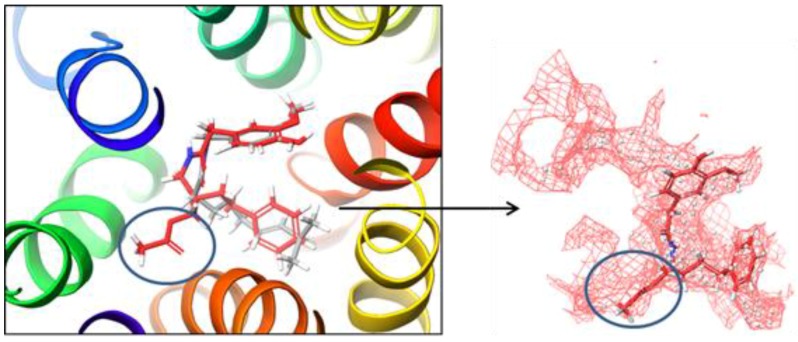
Binding mode of capsaicin (grey) and lead compound CID 44330438 (red) to the NorA efflux pump ligand binding site.

The lead compound proposed in this study, CID 44330438, could have the potential to restore the efficacy of the drug ciprofloxacin by inhibiting the NorA efflux pump. The CID 44330438–NorA complex revealed another region, between helices V (green) and VIII (orange) (Figure 4), that could provide additional substrate specificity. The compound is located on the cytoplasmic side, positioned close to the hydrophobic core, which may play a role in defining the topology of the inhibitor. The residue Val^22^, which is mapped onto helix I, may be important for drug recognition in a fashion that resembles the role of Val^17^ in the EmrD protein (see Figure 4) [22]. Based on the NorA structure model, we speculate that the inhibitor diffuses across the inner membrane together with the antibiotic, and that the inhibitor could be positioned near the cytoplasmic side, thus avoiding the efflux of the drug. 

From a pool of 620 compounds, only a few presented scoring functions that defined them as hit molecules. This finding provides some clues about the probability of identified substrates within the hit molecules. Nevertheless, a more detailed study of the mechanistic aspects of the interactions warrants further investigation to establish whether the mode of binding of hit molecules and substrates is similar. To this end, new direct structural information at the atom resolution of efflux pumps is urgently needed to understand proteins in their biological roles [26].

## 3. Materials and Methods

### 3.1. Protein Modeling: Preparation of NorA Efflux Pump

The NorA amino acid sequence from *Staphylococcus aureus* (UniProt Q5HHX4) [21] was used for comparative modeling, using the Swiss Model server [27]. The protein EmrD efflux pump (SMS) from *Escherichia coli* (available in Protein Data Bank, under ID 2GFP) [22] was identified by the Swiss Model as the closest homolog of NorA. Thus, the EmrD crystal structure was used to prepare the target 3D structure model, which then was optimized using the “Protein Preparation Wizard” tool integrated in Maestro (Schrödinger, LLC, New York, NY, USA) [28]. The quality of the NorA 3D structure model was assessed using the SAVES v5.0 server [23], which validates 3D structures using the programs Verify 3D, Errat, Prove, Procheck, and Whatcheck, and with the program Coot. All of these programs demonstrated that no further modification to the 3D structure model—such as new rounds of structure refinement—was required. Missing hydrogen atoms were added, assuming the standard protonation state of titratable residues. Subsequently, the Schrödinger’s SiteMap’s algorithm [29] was used to identify the binding site of the protein. The grid file—which represents physical properties of a volume of the receptor—was set to the binding core of the protein, consisting of the residues Ile^19^, Ile^23^, leu^26^, Ile^135^, Ile^244^, Lys^44^, Gln^51^, Gln^248^, Gln^325^, Pro^27^, Phe^47^, and Trp^293^, using the receptor grid generation tools of Glide [30]. The size of the docked molecules was set to be within 15 Å. 

### 3.2. Procedure for Molecular Docking Simulation

The chemical structures of the new compounds were retrieved from PubChem using the capsaicin chemotype as the lead structure. A PubChem dataset of 673 compounds with >0.8 Tanimoto similarity to capsaicin, and which complied with Lipinsky’s rules, was extracted [31,32]. Furthermore, the SwissADMET [33] server was used to evaluate in silico PAINS rules, which reduced the number of compounds to 620. All these molecules were chosen for docking studies. Low-energy three-dimensional conformations of the molecules were prepared using the LigPrep module of the Schrödinger package. Additionally, the Epik software [34] was employed to predict pKa values in the pH range between 7.0 and 7.5, and to return all chemically sensible structures using Hammett and Taft methodology. All compounds were minimized using the OPLS3 force field implemented in Maestro [35]. 

Molecular Dinamic (MD) simulations of protein-inhibitors and protein–substrate complexes were carried out using the Schrödinger bioinformatics suite. To achieve this, molecular docking was performed using the high-throughput virtual screening (HTVS) Glide-dock [36,37] module. Ligand flexibility was used to explore an arbitrary number of torsional degrees of freedom, in addition to the six spatial degrees of freedom spanned by the translational and rotational parameters. Ligand poses generated in such a way were run through a series of hierarchical filters to evaluate ligand interactions with the receptor. Docking score, glide gscore, glide emodel, ionization penalty, and topological polar surface area (TPSA) were used to select the docking poses [38]. 

The ADME/Tox profile of the best molecules identified by the HTVS was calculated in silico. For this purpose, a set of 34 physicochemical descriptors was computed using QikProp version 3.5 integrated in Maestro (Schrödinger, LLC, New York, NY, USA). The QikProp descriptors are depicted in Table S3. 

**Scheme 2:**
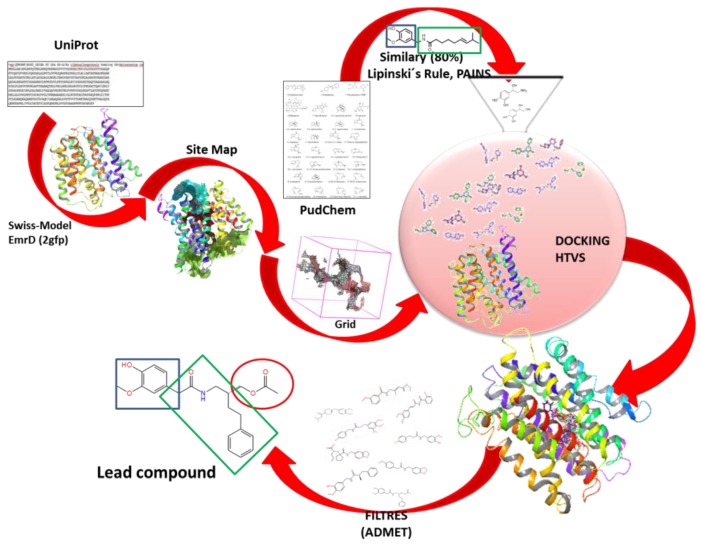
Computational protocol used for the prediction of new potential inhibitors of the NorA efflux pump from *S. aureus*.

The computational protocol used in this study is shown in Scheme 2. The chemical structures of the novel candidate inhibitors of NorA shared more than 80% geometrical similarity to capsaicin. Using this strategy, 10 promising compounds were identified using HTVS-docking, including ADME/Tox filters.

## 4. Conclusions

This study integrated an approach that combined homology modeling, molecular docking, and structure-based virtual screening for the prediction of one potential new inhibitor (CID 44330438) of NorA. The aromatic component of the small size inhibitor and its conformational pose seem to be important contributors to stabilizing the interaction with the binding core of the NorA protein. The in silico pharmacokinetics and toxicity (Lipinski’s rules of five, PAINS, and ADMET) of the compound were assessed and found to be satisfactory [39]. Furthermore, details of the mode of binding of the new potential inhibitor CID 44330438 to NorA provides new insight into the molecular mechanism of inhibition of MFS efflux pump transporters. Taken together in a broader context, our results can inform the design of novel MFS efflux pump inhibitors with increased efficacy, and be used to develop innovative therapies for the treatment of *S. aureus* infections, particularly those caused by multidrug-resistant strains.

## Figures and Tables

**Table 1:** Binding energy (kcal/mol) of the lead compounds to the NorA efflux pump.

CID PubChem	Residues Implicated in the Interaction	Docking Score (kcal/mol)
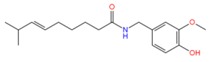 Capsaicin (1548943)	Hydrophobic: Phe^16^, Ile^19^, Ile^23^, Ile^244^ Pi–Pi stacking: Phe^47^, Trp^293^	−7.19
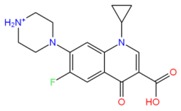 Ciprofloxacin (2764)	Hydrophobic: Val^22^, Val^44^, Ile^23^, Leu^26^, leu^43^, Ala^46^ Pi–Pi stacking: Phe^47^	−6.80
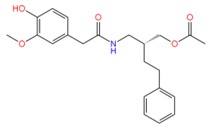 44330438	Hydrophobic: Val^22^, Ile^23^, Ala^46^, Ala^49^ Pi–Pi stacking: Phe^47^	−8.14
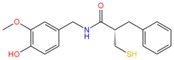 14557750	Hydrophobic: Ile^19^, Ile ^23^, Val^22^, Val^44^, Leu^26^, leu^43^, Ala^46^ Pi–Pi stacking: Phe^47^	−8.02
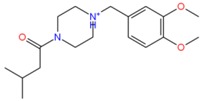 742523	Hydrophobic: Met^103^, Leu^43^, leu^40^, leu^26^, Ile^23^, Pro^27^ Pi–Pi stacking: Phe ^47^	−7.77
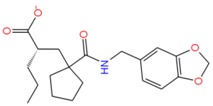 11516039	H-bond: Thr^223^ Hydrophobic: Leu^43^, Leu^26^, Val^44^, Val^22^, Ala^46^, Phe^47^, Phe^16^	−7.65
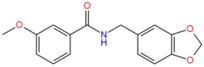 790127	Hydrophobic: Tyr^228^, Pro^27^, Leu^26^, Ile^23^	−7.45
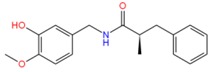 5459532	Hydrophobic: Val^44^, Val^22^, Leu^43^, leu^40^, Leu^26^, Ile^23^	−7.40
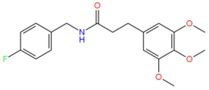 2107051	Hydrophobic: Tyr^292^, Met^296^, Met^109^, Ile^19^, Ile^23^ Pi–Pi stacking: Phe^47^, Trp^293^ H-bond: Trp^293^	−7.37
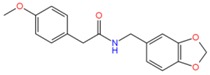 754514	Hydrophobic: Val^44^, Val^22^, Leu^43^, Leu^26^, Ile^23^, Ile^19^ Pi–Pi stacking: Phe^47^	−7.37
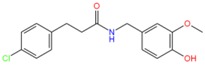 44316847	Hydrophobic: Pro^27^, Leu^26^, Ile^23^, Ile^19^, Trp^293^, Tyr^292^	−7.33
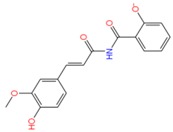 2175449	Hydrophobic: Tyr^131^, Tyr^292^, Ile^244^, Ile^19^, Ile^23^, Ile^240^, Met^103^, Val^44^ Pi–Pi stacking: Phe^47^	−7.20

**Table 2:** Computational prediction of drug likeness properties of potential new lead compounds.

CID PubChem ID	HBA	HBD	QPlogherg	MW	QPlogS	QPlog Kp	QPlog Khsa	QPP Caco	PHOA
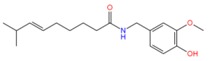 Capsaicin (1548943)	3	2	−3.76	305.1	−4.08	−1.90	0.14	1788.6	100
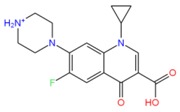 Ciprofloxacin (2764)	7	2	−3.43	331.1	−3.79	−6.48	0.01	13.3	49
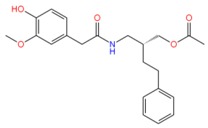 44330438	5	2	−4.04	385.1	−4.55	−2.04	0.08	504.9	95
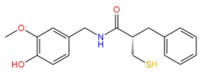 14557750	4	3	−4.12	331.1	−4.09	−1,31	0.11	1001.8	100
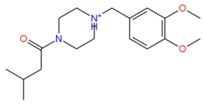 742523	4	0	−4.02	320.2	−1.99	−3,29	−0.45	964.2	93
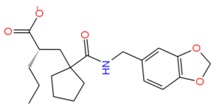 11516039	5	2	−1.26	361.1	−3.48	−2.27	−0.29	193.7	86
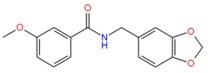 790127	4	1	−4.99	285.1	−3.43	−1.04	−0.03	3710.8	100
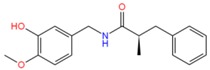 5459532	3	2	−3.97	299.1	−3.64	−1.54	−0.06	1907.6	100
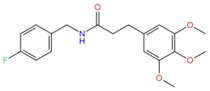 2107051	5	1	−3.18	347.1	−4.14	−1.04	0.18	2653.4	100
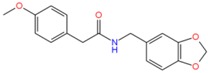 754514	4	1	−3.19	299.1	−2.34	−1.22	−0.26	1725.0	100
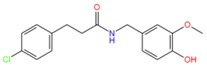 44316847	3	2	−4.01	319.0	−3.51	−1.41	−0.06	1016.0	100
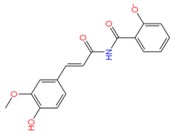 2175449	5	3	−5.95	313.0	−4.43	−3.31	0.19	156.1	82

1548943: capsaicin; 2764: ciprofloxacin. HBA: hydrogen bond acceptor; HBD: hydrogen bond donor; QPlogS: predicted aqueous solubility (−6.5/0.5); QPlogKp: predicted skin permeability (−8.0/−1.0); QPlogKhsa: prediction of binding to human serum albumin (−1.5/1.5); PHOA: percent human oral absorption (>80% is high and <25% low); QPPCaco: apparent Caco-2 cell permeability in nm/s (intestinal drug permeability) (<25 poor, >500 excellent); QPlogHERG: cHERG K+ channel blockage (log IC50) (concern below −5, range of 95% of drugs). The selected compounds present a null Pan Assay Interference Compounds (PAINS) profile.

## Supplementary Materials

The following are available online at https://www.mdpi.com/2079-6382/8/1/25/s1, Figure S1: Pairwise alignment of NorA and EmrD MFS transporter proteins, Figure S2: Quality of the NorA model, Figure S3: Location of binding sites of NorA model, Table S1: Hydropathy analysis of Quinolone resistance protein NorA from *S. aureus* and EmrD from *E. coli*, Table S2: Conserved residues for EmrD and NorA efflux pump proteins, and Table S3: The 20 best lead compounds obtained by QikProp.
