# Supplementary material for: A Molecular Modeling Approach to Identify Novel Inhibitors of the Major Facilitator Superfamily of Efflux Pump Transporters
Source: Antibiotics (Basel). 2019 Mar 15;8(1):25. doi: 10.3390/antibiotics8010025 (PMC6466568; doi:10.3390/antibiotics8010025)
Supplement: Supplementary file 1 [file antibiotics-08-00025-s001.pdf]

**Supplementary Materials**

# A molecular modelling approach to identify novel inhibitors of the major facilitator superfamily of efflux pump transporters

**Table S1**.- Topological domain of MFS transporters (NorA from *S. aureus* and EmrD from *E. coli*).

| Topological domain | NorA model | | EmrD (pdb ID:2gfp)*[22] | |
|---|---|---|---|---|
| | Stard | End | Stard | End |
| Helix I | 5 | 25 | 9 | 29 |
| Helix II | 42 | 62 | 47 | 67 |
| Helix III | 69 | 89 | 74 | 94 |
| Helix IV | 99 | 119 | 96 | 116 |
| Helix V | 129 | 149 | 135 | 183 |
| Helix VI | 157 | 167 | 163 | 176 |
| Helix VII | 201 | 221 | 213 | 233 |
| Helix VIII | 239 | 259 | 243 | 263 |
| Helix IX | 269 | 289 | 277 | 297 |
| Helix X | 293 | 313 | 299 | 319 |
| Helix XI | 331 | 351 | 330 | 350 |
| Helix XII | 355 | 375 | 365 | 385 |

**Figure S1**.- Pairwise alignment of NorA and EmrD MFS transporter proteins (Swiss-Model, https://swissmodel.expasy.org/).

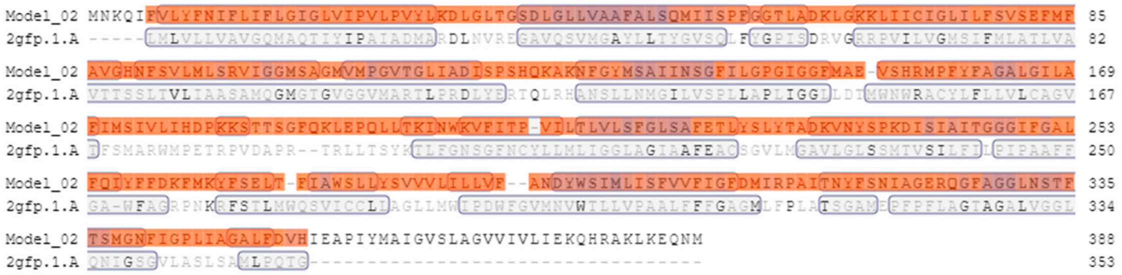

**Figure S2.-** Quality of the NorA model

A) Ramachandran plot: Residues in favorable regions 84%, in allowed 12,3% and in disallowed regions 0.7% (Ala$^{316}$, Leu$^{28}$ and Gly$^{61}$). Good quality NorA model with the residues >90% in the most favored regions. (Plot done with Maestro suit). Red: Favorable, Yellow: Allowed, white: disallowed.

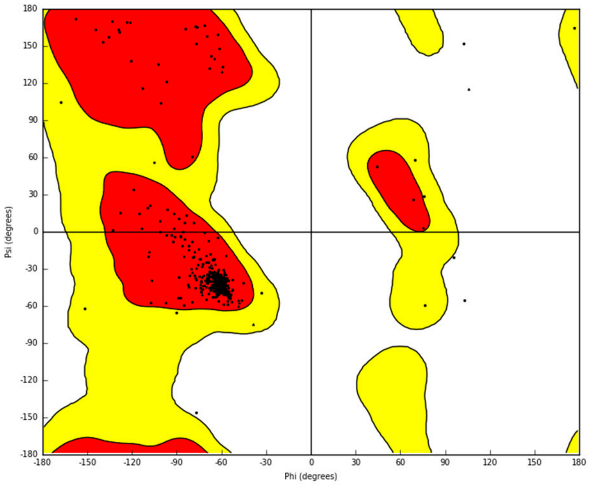


B) 3D profiles of NorA model: The non-bonded interactions between various types of atoms were computed with ERRAT2 program which showed an overall quality factor of **79,12.

*On the error axis, two lines are drawn to indicate the confidence with which it is possible to reject regions that exceed that error value. **Expressed as the percentage of the protein for which the calculated error value falls below the 95% rejection limit. Good high resolution structures generally produce values around 95% or higher. For lower resolutions (2.5 to 3A) the average overall quality factor is around 91%.

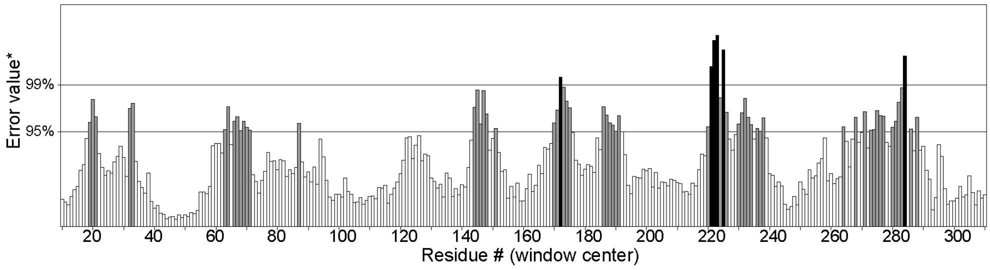


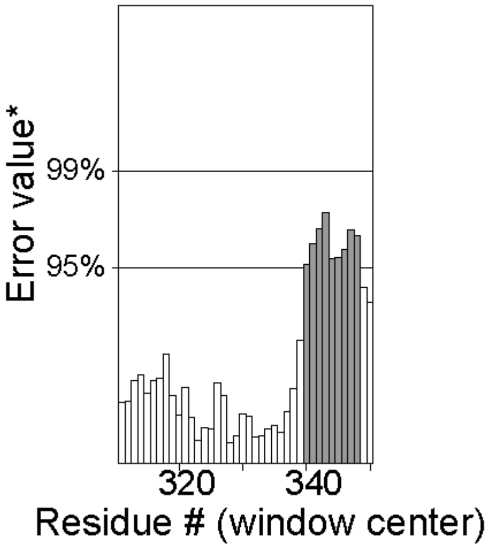

**<u>Figure S3.-</u>** Locate binding sites of NorA model.

Red: binding core ( D-II), Blue: periplasmic side (D-I) and Green: Cytoplasmatic side (D-III).

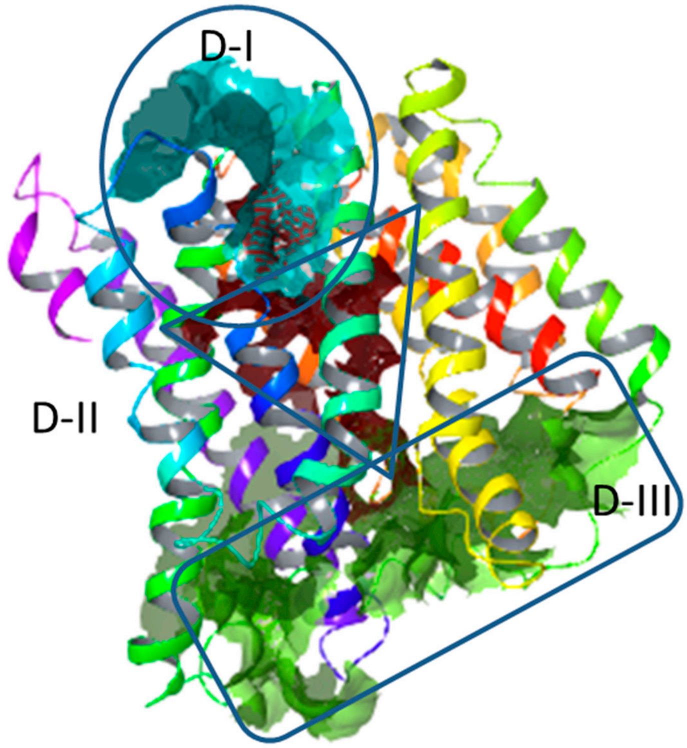


**<u>Figure S4</u>.-** Stereoimage view of the hydrophobic core of NorA (Phe$^{47}$, Tyr$^{292}$, Tyr$^{131}$ and Trp$^{293}$ residues)

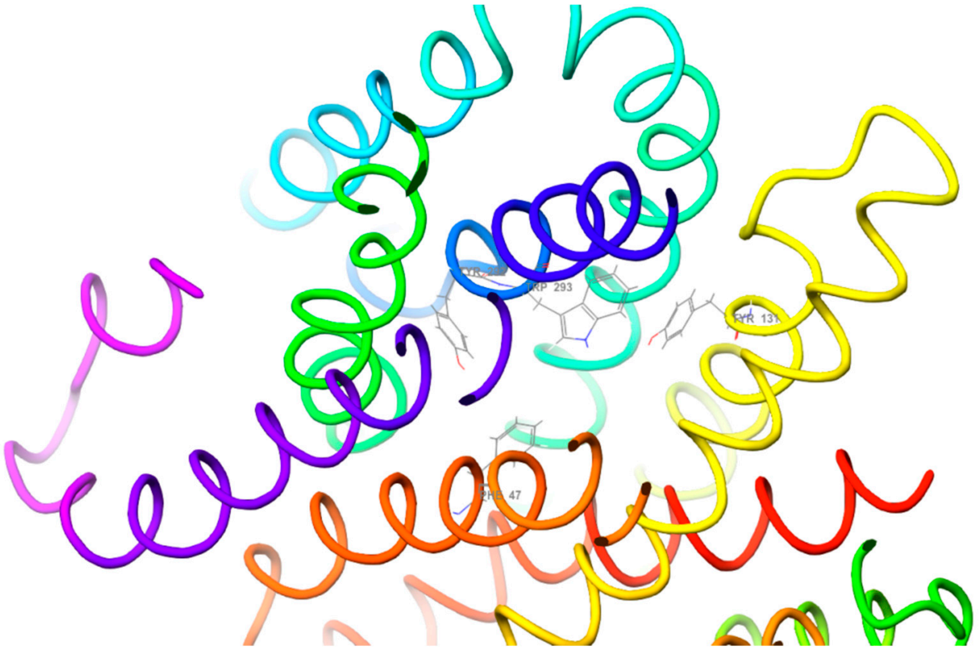

**Table 2.** Conserved residues for EmrD and NorA efflux pump proteins.

| Cavities | Amino Acid | EmrD efflux pump | Amino Acid | NorA efflux pump |
|---|---|---|---|---|
| Binding Site | Ile | 28 | Ile | 23 |
| | Ile | 217 | Ile | 209 |
| | Tyr | 52 | Phe | 47 |
| | Trp | 300 | Trp | 293 |
| | Phe | 249 | Ile | 244 |
| Cytoplasmic side | Arg | 118 | Thr | 113 |
| Periplasmic side | Thr | 25 | Ile | 19 |
| | Asp | 33 | Asp | 32 |
| | Glu | 227 | Glu | 222 |

**Table S3.-** Computational prediction of drug likeness properties of potential new 18 lead compounds. Yellow: Compounds do not present good druglikeness.

| CID PudChem | HBA | HBD | MW | QPlogS | QPlogHERG | QPPCaco | QPlogKp | QPlogKhsa | Percent Human Oral Absorption |
|---|---|---|---|---|---|---|---|---|---|
| **Capsaicin** | 3 | 2 | 305 | −4.08 | −3.76 | 178,6 | −1.90 | 0.14 | 100 |
| **Ciprofloxacin** | 7 | 2 | 331 | −3.79 | −3.43 | 13 | −6.48 | 0.01 | 49 |
| **44330438** | 5 | 2 | 385 | −4.55 | −4.05 | 505 | −2.05 | 0.08 | 95 |
| **2900500** | 3 | 1 | 327 | −4.75 | −4.49 | 3400 | −0.39 | 0.31 | 100 |
| **14557750** | 4 | 3 | 331 | −4.09 | −4.12 | 1002 | −1.31 | 0.11 | 100 |
| **2107051** | 5 | 1 | 347 | −4.02 | −3.65 | 2406 | −0.94 | 0.10 | 100 |
| **1740989** | 3 | 1 | 327 | −3.57 | −3.56 | 2494 | −0.58 | 0.20 | 100 |
| **822484** | 3 | 1 | 297 | −2.98 | −3.49 | 2666 | −0.69 | −0.06 | 100 |
| **742523** | 4 | 0 | 320 | −1.99 | −4.02 | 964 | −3.29 | −0.45 | 93 |
| **11516039** | 5 | 2 | 361 | −3.48 | −1.27 | 194 | −2.27 | −0.29 | 86 |
| **790127** | 4 | 1 | 285 | −3.43 | −4.99 | 3711 | −1.04 | −0.03 | 100 |
| **877843** | 3 | 1 | 269 | −2.83 | −3.86 | 2892 | −0.71 | −0.24 | 100 |
| **5459532** | 3 | 2 | 299 | −3.64 | −3.97 | 1097 | −1.54 | −0.06 | 100 |
| **1909740** | 3 | 1 | 313 | −3.87 | −3.69 | 3132 | −0.64 | 0.12 | 100 |
| **2107051** | 5 | 1 | 347 | −4.14 | −3.18 | 2653 | −1.04 | 0.18 | 100 |
| **754514** | 4 | 1 | 299 | −2.34 | −3.20 | 1725 | −1.22 | −0.26 | 100 |
| **44316847** | 3 | 2 | 320 | −3.51 | −4.01 | 1016 | −1.41 | −0.06 | 100 |
| **742523** | 4 | 0 | 320 | −2.02 | −4.12 | 835 | −3.35 | −0.43 | 92 |
| **44330438** | 5 | 2 | 385 | −4.34 | −4.33 | 250 | −2.42 | 0.08 | 89 |
| **288409** | 3 | 1 | 299 | −3.51 | −3.61 | 3457 | −0.57 | −0.01 | 100 |
